# Differentiation of subgenomes in StY-genomic species of the genus Elymus (Triticeae, Poaceae) from the territory of Russia according to sequencing data of the nuclear gene GBSS1 (waxy)

**DOI:** 10.18699/vjgb-26-60

**Published:** 2026-07

**Authors:** A.V. Agafonov, E.V. Shabanova (Kobozeva), A.A. Bondar, O.V. Dorogina

**Affiliations:** Central Siberian Botanical Garden of the Siberian Branch of the Russian Academy of Sciences, Novosibirsk, Russia; Central Siberian Botanical Garden of the Siberian Branch of the Russian Academy of Sciences, Novosibirsk, Russia; Institute of Chemical Biology and Fundamental Medicine of the Siberian Branch of the Russian Academy of Sciences, Novosibirsk, Russia; Central Siberian Botanical Garden of the Siberian Branch of the Russian Academy of Sciences, Novosibirsk, Russia

**Keywords:** molecular markers, phylogeny, subgenome, taxonomy, Elymus, Pseudoroegneria, молекулярные маркеры, филогения, субгеном, таксономия, Elymus, Pseudoroegneria

## Abstract

According to the latest data, 55 species of the genus Elymus are distributed in Russia, carrying three subgenomic combinations (StH, StY, and StHY). Among them, the StY-genomic group comprises only 10 species, with varying degrees of understanding of their evolutionary relationships. Ancestral taxa of the genus Pseudoroegneria donated the St subgenome to all modern species of the genus Elymus. The StY-genomic group of species possesses an additional Y subgenome of unknown origin, which, according to some data, is close to the St subgenome. We studied the phylogenetic relationships between StY-genomic species from Russia, five species of the genus Pseudoroegneria, and five species of the genus Hordeum from the NCBI GenBank by comparing the nucleotide sequences of the nuclear gene GBSS1 from exons 9 to 14. One of the main objectives was a comparative analysis of phylogenetic patterns based on (i) more conservative exons and (ii) introns that do not encode the amino acid sequences of the enzyme. All gene variants from the St subgenomes of the studied species are divided into three clusters according to three marker groups of nucleotide sequence of clones in the genus Pseudoroegneria: Central Asian (St1) with the reference species P. strigosa, North American (St2) with the marker species P. spicata, and Middle Eastern (St3) with two species, P. tauri and P. libanotica, to which the Eastern European species P. stipifolia gravitates. The H subgenome, originating from ancestral taxa of the genus Hordeum (Critesion), was not detected in any of the studied species. The cluster of Y subgenome in the “introns” variant is generally visually less differentiated than in the “exons” variant. This fact contradicts the established notion that gene regions responsible for the synthesis of enzymatic molecules are more conserved. Among the most notable characteristics in the comparison of nucleotide sequences is the presence of a special Far Eastern race of E. gmelinii with St3 sequences instead of St1 and the location of the sequences of all clones of the North Kazakhstan accession E. fedtschenkoi KSA-0935 in the St2 cluster instead of St3

## Abstract (ru)

По последним данным, на территории России распространены 55 видов рода Elymus, которые являются носителями трех субгеномных комбинаций: StH, StY и StHY. Среди них StY-геномная группа насчитывает всего 10 видов с разной степенью изученности эволюционных связей. Предковые таксоны рода Pseudoroegneria были донорами субгенома St для всех современных видов рода Elymus. StY-геномная группа видов обладает дополнительным субгеномом Y с неизвестным происхождением, который по некоторым сведениям близок к субгеному St. Нами изучены филогенетические взаимоотношения между StY-геномными видами с территории России, пятью видами рода Pseudoroegneria и пятью видами рода Hordeum из Генбанка NCBI путем сравнения нуклеотидных последовательностей ядерного гена GBSS1 с 9-го по 14-й экзон. Проведен анализ участков более консервативных экзонов в сравнении с интронами, которые не кодируют аминокислотные последовательности фермента. Все варианты гена из субгеномов St у изученных видов подразделяются на три кластера сообразно трем маркерным группам нуклеотидных последовательностей клонов рода Pseudoroegneria: центральноазиатской (St1) с реперным видом P. strigosa, североамериканской (St2) с маркирующим видом P. spicata и ближневосточной (St3) с двумя видами – P. tauri и P. libanotica, к которым тяготеет восточноевропейский вид P. stipifolia. Ни у одного из изученных видов субгеном Н, имеющий происхождение от предковых таксонов рода Hordeum (Critesion), не обнаружен. Что касается Y-субгенома в варианте «интроны», то в целом этот кластер менее дифференцирован, чем в варианте «экзоны». Данный факт противоречит сложившимся представлениям о большей консервативности генных участков, ответственных за синтез ферментных молекул. Среди наиболее примечательных характеристик в сравнении нуклеотидных последовательностей следует отметить наличие особой дальневосточной расы E. gmelinii с последовательностями St3 вместо St1 и расположение последовательностей всех клонов северо- казахстанского образца E. fedtschenkoi KSA-0935 в кластере St2 вместо St3.

## Introduction

The genus of perennial allopolyploid herbs Elymus L. is the largest in the tribe Triticeae Dumort. (Poaceae Barnh.). There are between 150 and 200 species worldwide with different genomic constitutions, all of which are considered to be unified by the St subgenome. According to generalized data, this subgenome is present in different taxa in at least eleven combinations with six other subgenomes of the ancestral taxa of the tribe: H, Y, P, W, Ns, and Xm (Tan et al., 2024). In recent decades, there has been a trend toward splitting the genus Elymus s. l. into eight independent allopolyploid genera, including Elymus s. str. (StH), Roegneria C.Koch (StY), Douglasdeweya C. Yen, J.L. Yang & B.R. Baum (StP), Campeiostachys Drobow (StYH), Kengyilia C. Yen & J.L. Yang (StYP), Anthosachne Steudel (StYW), Pascopyrum Á. Löve (StHNsXm), and Connorochloa Barkworth, S.W.L. Jacobs & H.Q. Zhang (StYWH).

Since the genus belongs to the tertiary gene pool (GP-3) of major cereal crops, its representatives have often been included in detailed cytogenetic studies (Dewey, 1984). In recent decades, the cytogenetic method of determining the genomic constitution of species has been supplemented and even superseded by more technologically advanced DNA sequencing methods (Mason-Gamer, 2001; Baum et al, 2003). Researchers are shifting their focus to analyzing the origins and phylogenetic relationships between basic subgenomes using molecular genetic methods.

The main diversity of species in the StY genomic group is concentrated in China, Korea, and Japan. A number of contradictory data on the phylogenetic relationships between the St and Y subgenomes have been published. Specifically, in the search for a Y subgenome donor, hybridization was conducted between diploid Hordeum L. species (carriers of the H subgenome) and StY-genomic Asian Elymus species. Triploid hybrids were characterized by a relatively low level of chromosome pairing in metaphase I, demonstrating extremely low homology between the H, St, and Y haplomes and, consequently, the absence of the H haplome in these Elymus species (Lu, von Bothmer, 1990). In addition, based on the analysis of chromosomal pairing in hybrids, a low relationship between the St and Y subgenomes was shown (Sakamoto, 1964; Lu, von Bothmer, 1989).

The nuclear ribosomal internal transcribed spacer (ITS) and chloroplast intergenic spacer trnL-F sequences were analyzed in 457 Elymus accessions containing diverse genomes. The results confirmed that the St, H, P, and W subgenomes of polyploid Elymus were introduced from the genera Pseudoroegneria (Nevski) A. Löve, Hordeum, Agropyron Gaertn., and Australopyrum (Tzvelev) A. Löve, respectively, but that the St and Y subgenomes phylogenetically trace back to a common ancestor (Liu et al., 2006).

In another study, one of the RAPD markers specific to the Y subgenome was converted into a sequence-tagged site (STS) marker. This modified STS marker confirmed the presence of a Y subgenome in 42 accessions of StY-genomic Elymus species (Okito et al., 2009). To identify possible donors of the Y subgenome, 43 accessions of the diploid Pseudoroegneria species with the St subgenome were analyzed using the STS marker. The authors concluded that some StY-genomic Elymus species harbored this particular variant of the Y subgenome, shared with the St genome of Pseudoroegneria, that is, a precursor subgenome designated StY.

Later, the single-copy nuclear gene encoding elongation factor G (EF-G) was analyzed among 28 accessions of polyploid Elymus species and 45 accessions of diploid species of the tribe Triticeae (Sun, Komatsuda, 2009). The data supported the hypothesis that the Y subgenome arose in a diploid species but had a different origin compared to the St subgenome. This conclusion was supported by the single-copy nuclear RNA polymerase II gene (RPB2) from 58 accessions of Pseudoroegneria and Elymus species (Yan et al., 2011).

Further, based on the topology of the phylogenetic tree using chloroplast noncoding DNA sequences from 56 accessions of nine polyploid Elymus species, the authors showed that the St and Y subgenomes did not originate from the same donor, and that the Y subgenome likely originated from the H genome of Hordeum species (Song et al., 2015).

In a recent report, the following important conclusions were made based on the analysis of sequence data from three nuclear DNA regions (Acc1, DMC1, and Pgk1) and three chloroplast DNA regions (nrITS) (matK, rbcL, and trnL-trnF) (Pan et al., 2025):

1. The Triticeae polyploid species, which consist of different genome types, should be treated as a distinct genera. 2. Some polyploid species with the St genome have undergone independent allopolyploidization events in different regions of distribution. 3. The Y genome originated from an unknown or extinct diploid species closely related to Peridictyon sanctum (Janka) Seberg, Fred. & Baden, which is distributed on the Balkan Peninsula (Xp genome).

In this regard, we are inclined to support the view expressed three years earlier, which proposed an autotetraploid origin of the Y subgenome through recurrent hybridization (Liu et al., 2022). According to these data, the complex St genomes of the ancestors of the genus Pseudoroegneria in the polyploid state may have gained greater opportunities for intraspecific differentiation through repeated and back-hybridization. As a result, modified variants of the St genomes in some lineages evolved into intermediate StY genomes.

Thus, one likely scenario is the origin of the modern Y subgenome from taxa of the genus Pseudoroegneria carrying ancestral St subgenome variants, while the origin of the Y subgenome from the relatively distant H and Xp genomes appears unlikely.

As repeatedly demonstrated in the work of Dr. R. J. Mason- Gamer’s research group from 1998 to 2024, the nuclear gene encoding granule-associated starch synthase 1 (GBSS1, waxy) can serve as an effective genetic marker of macro- and microevolutionary relationships between taxa of different levels. The gene’s schematic was first presented using Zea mays L. as an example (Mason-Gamer et al., 1998), but information on its specificity among North American species of the genus Elymus was published three years later (Mason-Gamer, 2001).

We confirmed the feasibility of using this gene as an indicator of microevolutionary processes among species of the genus Elymus (Agafonov et al., 2019, 2024a, b). It is generally accepted that intron sequences are not subject to strict evolutionary selection; therefore, they can accommodate numerous intermediate microevolutionary events (substitutions, insertions, etc.) that occurred during the evolution of subgenomes. Moreover, the biological function of introns, as one of the constituent parts of the nucleotide sequences of a number of functional genes, appears to be quite important in relation to the regulation of gene expression (Koonin, 2006).

According to the latest data (Tzvelev, Probatova, 2019), 55 species of the genus Elymus are distributed in Russia. These are allotetra- and allohexaploids with the StStHH, StStYY, and StStHHYY genomes (Agafonov et al., 2020). Among them, the StY genome group comprises only 10 species, with varying degrees of understanding of their evolutionary relationships.

Previously, we proposed new combinations of taxa within this group in a broader sense, specifically for E. pendulinus (Nevski) Tzvelev s. l. (Kobozeva, Agafonov, 2015) and E. ciliaris (Trin.) Tzvelev s. l. (Agafonov et al., 2021; Shabanova (Kobozeva), Agafonov, 2023). Species boundaries are delineated based on the “principle of microevolutionary complexes”, that is, a set of closely related taxa that defines the phylogenetic unity of a species and meets other criteria. The base species includes intraspecific taxa based on their relationships established by reproductive and molecular genetic criteria. This principle can ensure methodological synergy between the fundamental principles of traditional taxonomy, on the one hand, and experimental biology incorporating modern approaches and methods, on the other. As a result, the total number of StY-genomic species in Russia has been reduced to seven.

This study aimed to trace the evolutionary relationships between the St subgenomes and to assess the position of the Y subgenome on the evolutionary tree among StY-genomic Elymus species from Russia by comparing the nucleotide sequences of GBSS1 gene fragments of exons from 9 to 14. Specifically, the goal was to identify differences in the phylogenetic picture based on the sequences of more conserved exons compared to introns not involved in the final synthesis of enzyme molecules.

## Materials and methods

Plant material. The study included 32 natural accessions of all StY-genomic species listed by N.N. Tzvelev and N.S. Probatova (2019). All of these accessions from our collection underwent morphological confirmation of taxonomic rank in experimental open-field plots and in a climate chamber to ensure compliance with modern taxon descriptions (Table S1)1.

Supplementary Materials are available in the online version of the paper: https://vavilov.elpub.ru/jour/manager/files/Suppl_Agaf_Engl_30_4.pdf


Two European species, E. caucasicus (C. Koch) Tzvelev and E. panormitanus (Parl.) Tzvelev, are not present in our living collection. The Central Asian species E. abolinii (Drobow) Tzvelev was additionally included in the comparative analysis, as we suspect it grows within Russia, but evidence is currently lacking

The GBSS1 gene sequences of five species of the genus Pseudoroegneria and five species of the genus Hordeum from the NCBI GenBank (URL: http://www.ncbi.nlm.nih.gov/ nuccore) were used as reference sequences (see the Table). Unlike the collection samples, the accession numbers of clones from NCBI are given in the dendrograms in front of the species names.

**Table 1.**
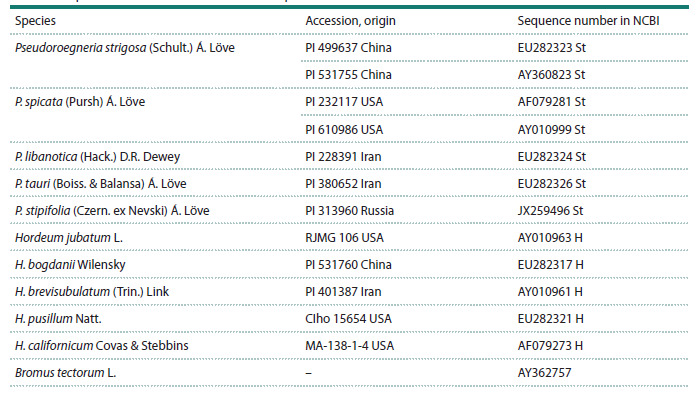
ScienReference species accessions and nucleotide sequence numbers taken from NCBI GenBank

Ancient representatives of these taxa were the donors of the St and H subgenomes, respectively, in modern species of the genus Elymus. Moreover, species of the genus Pseudoroegneria, confined to different ranges in Northern Eurasia, could have been the donors of the St subgenome in different species of the relatively young StY genome group of the genus Elymus.

For example, Pseudoroegneria strigosa was originally distributed in Central Asia and has also been recorded as native in Crimea and Greece (https://powo.science.kew.org/taxon/ urn:lsid:ipni.org:names:418876-1. First published in Taxon, 1980. 29: 168).

Pseudoroegneria spicata is native to the western part of the North American continent (https://powo.science.kew.org/ taxon/urn:lsid:ipni.org:names:1159330-2. First published in Taxon, 1980. 29: 168).

Pseudoroegneria libanotica and the following species are confined to territories in the Middle East (https://powo. science.kew.org/taxon/urn:lsid:ipni.org:names:942121-1. First published in P. Gustafson (ed.), Gene Manipulat. in Pl. Improv.: 1984: 272).

Pseudoroegneria tauri (https://powo.science.kew.org/taxon/ urn:lsid:ipni.org:names:914501-1. First published in Feddes Repert. 1984. 95: 445).

The fifth species, Pseudoroegneria stipifolia, has a more northerly range than the previous two (https://powo.science. kew.org/taxon/urn:lsid:ipni.org:names:914500-1. First published in Feddes Repert. 1984. 95: 445).

With regard to the use of molecular markers in the study of the genus Elymus, significant results were obtained by Dr. R. Mason-Gamer and co-workers (Helfgott, Mason- Gamer, 2004; Mason-Gamer, 2013; Mason-Gamer et al., 1998, 2010a, b). In particular, their studies showed that information on the nucleotide sequences of the low-copy gene “waxy” (granule-bound starch synthase 1 (GBSS1)) is consistent with cytogenetic data regarding the genomic constitution and evolutionary origin of North American (Mason-Gamer, 2001) and Asian (Mason-Gamer, 2010a) species of the genus Elymus.

The relatively small number of StY-genomic species in Russia makes it possible to trace the phylogenetic origin of the St subgenome in this group from the ancestral taxa of the genus Pseudoroegneria using data on the GBSS1 gene sequences in 6–8 clones per species accession.

Total DNA isotation, PCR amplification, cloning in a plasmid vector, DNA sequencing and construction of phylogenetic trees. Total DNA was isolated from 20 mg of dry green mass using the NucleoSpin Plant II Kit (Macherey- Nagel, Germany) according to the manufacturer’s standard protocol. GBSS1 gene fragment overlapping the region from exons 9 to 14 was obtained by PCR using a direct F-for primer (TGCGAGCTCGACAACATCATGCG, Mason-Gamer et al., 1998) and our modified M-bac-Alter1 (GGCGAGCGGYGCRATCTCSTSGCC) reverse primer.

The GBSS1 fragment was amplified under the conditions: 1X Q5 Reaction Buffer, 0.2 mM of each dNTP, 2.0 mM of free Mg2+, 0.3 μM of forward and reverse primers, about 0.4 ng/μL of genomic DNA, 8 u/mL of Q5 Hot Start II DNA Polymerase, with the addition of Betaine up to 1M and Q5-High GC Enchancer up to 1X. The temperature profile consisted from melting at 98 °C for 30 s, followed by 38 cycles of three stages (denaturation at 98 °C – 5 s; annealing at 69 °C – 10 s; elongation at 72 °C – 1 min), then completion at 72 °C – 2 min and storage at +4 °C. The fragments obtained were then cloned by ligation at the blunt ends into pJET2.1 vector using the CloneJET PCR Cloning Kit (Thermo Scientific, USA). By use of primers from the vector region (Jet_F and Jet_R) in PCR amplification of E. coli colonies grown on the plates (NEB stable) we have obtained fixed variants of the cloned GBSS1 gene. DNA fragments were then purified from PCR components by sorption on AMPure XP magnetic (Beckman Coulter, USA) and sequenced by Sanger method from both sides to assemble contigs and perform a subsequent analysis. From six to eight colony amplified DNA fragments of appropriate size containing separate GBSS1 clones for each genomic DNA sample were used for sequencing. The sequence AY362757 of Bromus tectorum L. was taken as an outgroup sample when constructing dendrograms

Multiple sequence alignment of the studied GBSS1 fragments was performed using the MUSCLE algorithm in the Unipro UGENE ver. 31.0 program (Okonechnikov et al., 2012). The aligned sequences were used to construct phylogenetic trees using the maximum likelihood (ML) method (Felsenstein, 1981). A series of dendrograms were constructed in the MEGA ver. 11.0.13 program (Tamura et al., 2021) using the two-parameter (2-parameter distance, K2P) evolutionary model of M. Kimura (1980) for GBSS1 fragments based on: a) exons separately; b) introns separately, as presumably more mobile sequences in microevolutionary terms. Bootstrap support values are indicated at the nodes. The nucleotide sequences of the clones of the GBSS1 gene regions we sequenced have been deposited into the GenBank (National Center for Biotechnology Information, NCBI) database. We use the abbreviation NSC to refer to them.

## Results

**Fig. 1.**
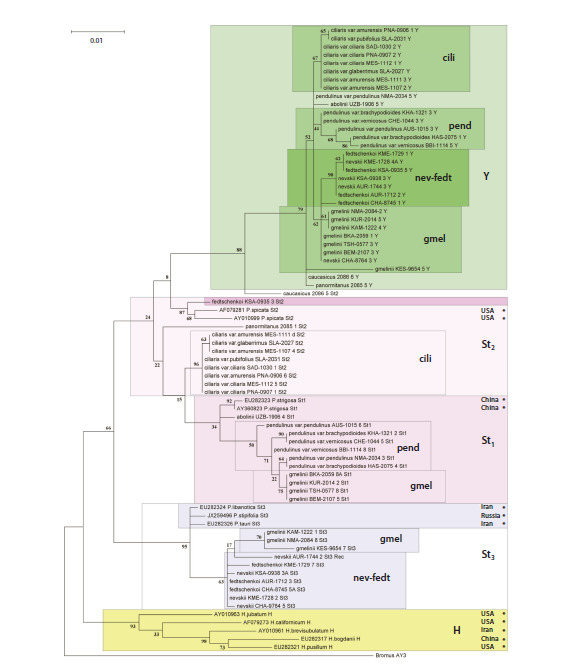
ML dendrogram constructed based on the analysis of GBSS1 gene sequences (exons 9–14 only) in the StYgenomic group of species from Russia compared to reference species. Asterisks indicate monogenomic carriers of the St and H subgenomes, as well as their origin. Bootstrap support values are shown at nodes

The dendrogram constructed using exon data without introns is shown in Figure 1. The following conclusions were drawn from its analysis.

1. All gene sequences in the St subgenome in the studied species are divided into three clusters according to three marker groups of the genus Pseudoroegneria: Central Asian (St1) with the reference species P. strigosa, North American (St2) with the marker species P. spicata, and Middle Eastern (St3) with two species: P. tauri and P. libanotica. The Eastern European species P. stipifolia gravitates toward the third cluster. 2. Conventionally, the first cluster St1, in addition to the marking sequences of P. strigosa, contains six NSCs of three varieties of E. pendulinus and four identical NSCs of E. gmelinii, of which three are Siberian and one is Far Eastern, as well as an NSC of the Central Asian species E. abolinii, which is widespread outside of Russia. 3. The North American cluster St2 contains a compact group of E. ciliaris varieties and a separate branch with two P. spicata sequences, along with the NSC of the North Kazakhstan accession E. fedtschenkoi KSA-0935_3 (highlighted in dark purple). It should be noted that only six St2 sequences were detected in this typical accession of E. fedtschenkoi. The genomic specificity of this accession will be described below. The sequence of the Crimean species E. panormitanus occupied a position between the two branches of this cluster with a bootstrap value of 22. 4. Somewhat unexpectedly, the third cluster St3 was differentiated, forming two main branches. One branch is formed by three marker species: P. tauri, P. libanotica, and P. stipifolia. The other branch comprises seven NSCs of E. nevskii– E. fedtschenkoi and three Far Eastern NSCs of E. gmelinii, two of which belong to Kamchatka accessions. It is unlikely that this division of E. gmelinii sequences between clusters St1 and St3 is random. Within this cluster, the sequence AUR-1744_2_Rec has separated the most. Nevertheless, we assigned the genomic formula St3 to all NSCs in this cluster. 5. The sequences of the Y subgenome are compactly located in a separate cluster, while predominantly preserving the species specificity of the large basic taxa E. ciliaris, E. pendulinus, E. gmelinii and the E. nevskii–E. fedtschenkoi complex. A single large clade was formed by the NSCs of the E. nevskii–E. fedtschenkoi complex, along with a group of six NSCs of E. gmelinii, which included the E. nevskii CHA-8764_3 sequence. The most isolated, as in the previous “exons” variant, were the Y-sequences of the European species E. caucasicus and E. panormitanus, as well as the NSCs of the Kamchatka accession E. gmelinii KES-9654_5. 6. An additional point worth noting is the location of the NSC of the Caucasian accession E. caucasicus H 2086, which, based on preliminary experiments, we classified as St2. This sequence occupies a position between the Y clusters and the St complex. Unfortunately, this accession has undergone several stages of exchange between gene banks and genotype collections, so there is no reason to consider its origin reliable.

The relatively close location of the Y subgenome cluster to the North American St2 subgenome cluster requires a separate historical analysis, since, according to some authors, the St and Y subgenomes originate from different ancestors (Sun, Komatsuda, 2009; Yan et al., 2011; Song et al., 2015). However, in general, the StY genomic group species from Russia have three evolutionary vectors, which is consistent with our understanding of microevolutionary complexes within the genus Elymus.

**Fig. 2.**
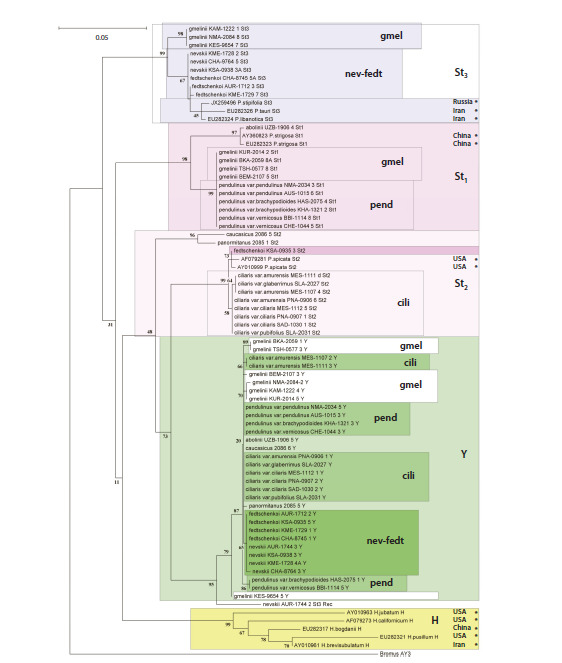
ML dendrogram constructed based on the analysis of GBSS1 gene sequences (introns 9–13 only) in the StY-genomic group of species from Russia compared to reference species. Asterisks indicate monogenomic carriers of the St and H subgenomes, as well as their origin. Bootstrap support values are shown at nodes.

When comparing the results obtained separately for exon (Fig. 1) and intron (Fig. 2) sequences, the following conclusions were reached.

1. In the “introns” dendrogram, all NSCs of E. pendulinus and four NSCs of E. gmelinii from the Central Asian group St1are located in a single branch close to the branch of two marker sequences of P. strigosa and the Uzbek accession of E. abolinii. Differences from the “exons” variant were not only evident in the bootstrap values. All NSCs of E. pendulinus and E. gmelinii were indistinguishable from each other in the St1 dendrogram. 2. A similar pattern of differences was evident in cluster St2, where the NSCs of the European E. caucasicus and E. panormitanus formed a separate branch. Within the E. ciliaris branch, two NSCs of E. ciliaris var. amurensis and a single NSC of E. ciliaris var. glaberrimus diverged slightly from five identical varieties. The sequence of the North Kazakhstan accession of E. fedtschenkoi KSA- 0935_3 maintained a close relationship with the marker sequences of P. spicata. 3. The group of NSCs St3, as in the “exons” variant, was divided into three branches, one of which included the Middle Eastern marker species of Pseudoroegneria, another united samples of the E. nevskii–E. fedtschenkoi complex (except for KSA-0935), and the third consisted of two Kamchatka and one Primorsky NSCs E. gmelinii. Overall, the most unexpected finding in the St sequence clusters was the identification of three E. gmelinii accessions as belonging to a distinct Far Eastern race of this species, with the St3 subgenome variant instead of the St1 variants characteristic of Siberia. However, one of the Primorsky NSC E. gmelinii BKA-2059 was located in the St1 cluster along with three Siberian accessions. Furthermore, all six NSCs studied from E. fedtschenkoi KSA-0935 (not shown in the dendrograms) were identified as belonging to the St2 group instead of St3 in both variants.

A study of the nucleotide composition of the introns of the studied species revealed that, despite the low variability of the Y subgenome sequences, one nucleotide substitution and one microdeletion in the NSC of E. gmelinii KES-9654_5 was sufficient for it to be clearly distinguished from all the others in the dendrogram.

H-subgenomic sequences were not identified in both variants of dendrogram construction among the studied accessions, which confirms the StY-genomic constitution of all species

**Fig. 3.**
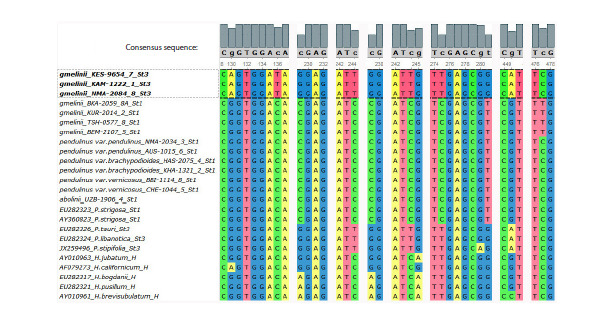
Differences in the sequences of exons 9–14 in three clones of Far Eastern E. gmelinii accessions carrying the St3 subgenomes and clones of E. gmelinii, E. pendulinus, and E. abolinii carrying the St1 subgenomes. Sequence fragments of marker clones of Pseudoroegneria are shown for comparison. The numbers above indicate the nucleotide positions in the aligned sequences.

**Fig. 4.**
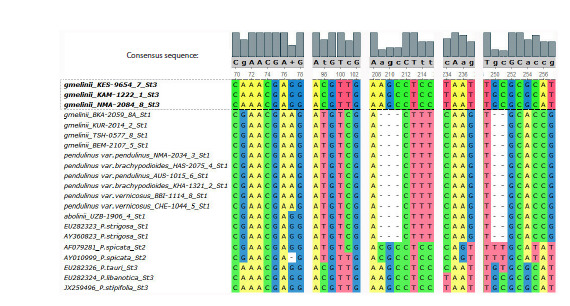
Differences in the sequences of introns 9–13 in three clones of Far Eastern E. gmelinii accessions carrying the St3 subgenomes and clones of E. gmelinii, E. pendulinus, and E. abolinii carrying the St1 subgenomes. Sequence fragments of marker clones of Pseudoroegneria are shown for comparison. The numbers above indicate the nucleotide positions in the aligned sequences.

As noted above, among the most notable characteristics in the tree topology is the presence of a distinct Far Eastern race of E. gmelinii with St3 NSCs instead of St1.To elucidate possible causes, we analyzed the sets of aligned sequences in critical regions of exons and introns. In Figures 3 and 4, the horizontal frames highlight fragments of three E. gmelinii NSCs located in St3 clusters in the exon and intron dendrograms, respectively, compared to the St1 sequences of other species and the St3 sequences of reference Pseudoroegneria accessions. The analysis revealed at least seven positions with nucleotides identical to the St3 marker clones in exon sequences, and also at least twelve positions in the intron sequences with complete differences from NSCs of St1 subgenome. In microevolutionary terms, this means that, having formed in ancestral genotypes, this allele became fixed in some Far Eastern populations of E. gmelinii and was transmitted from generation to generation virtually unchanged. However, to confirm the existence of a single Far Eastern race, it is necessary to define the presence of other distinctive characteristics, including signs of reproductive isolation.

## Discussion

Thus, the arrangement of subspecific taxa (varieties) in the two clustering variants was mixed, without any discernible specificity. This fact confirms the appropriateness of grouping species units within the StY genome group according to their phylogenetic relationships, based on previously formulated principles (Kobozeva et al., 2011, 2017; Kobozeva, Agafonov, 2015; Agafonov et al., 2021). Moreover, the Y subgenome showed a low level of variability, being historically young and having not accumulated evolutionarily significant transformations compared to the more ancient St1–St3 subgenomes. However, within the Y cluster, one can see evidence of separation of several microevolutionary complexes, such as the group of taxa from the St2 cluster of E. ciliaris and the group from the St3 cluster of E. nevskii–E. fedtschenkoi (highlighted in dark green in Figure 1).

Notably, the Y subgenome cluster in the “introns” variant is less variable than in the “exons” variant. This fact contradicts the established notion of greater conservation of gene regions responsible for the synthesis of enzymatic molecules. Outside the main branch from which most of the accessions originate lies the NSC of E. gmelinii – KES-9654_5_Y. The next most distant from this branch are two NSCs of E. pendulinus: HAS- 2075 and BBI-1114. All other species assemblages are located compactly and close to each other, including E. abolinii, E. caucasicus, and E. panormitanus. The NSC of E. nevskii, AUR-1744_2_St3_Rec, was located furthest on the dendrogram, intermediate between the Y and St2 clusters, possibly recombinant. The sequence of this clone had at least 11 single substitutions identical to nucleotides in the NSCs of the St1, St2, and H subgenomes, and received its Rec designation due to its location on the dendrogram within the “introns” part (Fig. 2).

**Fig. 5.**
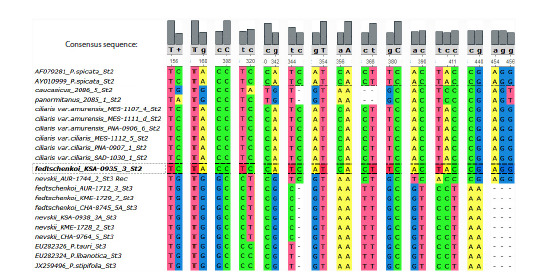
The horizontal frame shows the differences in the sequences of introns 9–14 in the NSC of E. fedtschenkoi KSA- 0935_3_St2 compared to those in the St3 genomic accessions of the E. nevskii–E. fedtschenkoi complex and the marker sequences of Pseudoroegneria. The numbers at the top indicate the nucleotide positions in the aligned sequences.

One notable result of the study was the location of all NSCs from the North Kazakhstan accession E. fedtschenkoi KSA- 0935 in the St2 cluster instead of St3. To clarify the subgenomic affiliation of the NSC E. fedtschenkoi KSA-0935_3_St2, aligned intron sequences in this NSC were compared with other St3 sequences of the E. nevskii–E. fedtschenkoi complex and reference Pseudoroegneria species (Fig. 5). Sequence analysis revealed that the NSC E. fedtschenkoi KSA-0935_3_St2 periodically carries substitutions identical to those in the NSC P. spicata St2 at positions distinct from the NSC St3 throughout its entire length. The possible origin of the St2 subgenome in E. fedtschenkoi can only be speculated about, given the North American range of the St2-genomic species P. spicata and the East Asian range of the St2-genomic species E. ciliaris s. l.

In our opinion, the group of StY-genomic species remains one of the least studied among the numerous species of the genus Elymus s. l. Species with the StH formula have been studied better than others for two main reasons: 1) historically, species from North America and Europe have primarily attracted the attention of researchers; 2) the accumulation of knowledge about the species and structural diversity of Central and East Asia has been slower due to the vast territories and numerous mountain ranges, which create a large number of isolated ecological niches and, consequently, a multitude of local races and distinct species.

Moreover, it is believed that StY-genomic species are found in North America only as adventitious species. Reliable finds are quite rare; herbarium specimens of three species containing the Y subgenome have been documented: E. ciliaris and E. semicostatus (Nees ex Steud.) Melderis, as well as E. tsukushiensis Honda, with the genomic formula StYH (Barkworth et al., 2007).

Currently, the number of experimental and systematic studies on the phylogeny of Asian species of the genus has increased dramatically due to the growing technological capabilities of the People’s Republic of China (Hu et al., 2013; Dong et al., 2015; Song et al., 2015; Lei et al., 2018; Liu et al., 2022; Pan et al., 2025), as well as the achievements of classical European and American scientific schools (Mason- Gamer, 2001; Leo et al., 2022, 2025; Mason-Gamer, White, 2024). In particular, the analysis of nuclear and chloroplast DNA showed that many Elymus species have multiple origins. The data suggest that hybridization and polyploidization were the main driving forces of evolution in increasing the biodiversity of the genus Elymus (Liu et al., 2006). This creates difficulties in resolving evolutionary relationships even when using high-throughput methods with a large number of genetic markers.

Furthermore, phenotypic plasticity, a small number of reliable morphological diagnostic characters, and a large number of taxa with the St subgenome in their genotypes complicate taxonomic analysis in practice (Leo et al., 2025). Nevertheless, an increasing number of new species are being described using molecular genetic data (Sha et al., 2024; Zhang et al., 2024; Alieva et al., 2025). Our results revealed signs of internal genetic divergence in E. gmelinii, the most widespread of all StY-genomic species in Russia.

The data obtained revealed a relationship between the conventional nomenclature of the St subgenome (St1, St2, and St3) and a specific group of species carrying a specific subgenome. Furthermore, similarities were found in fragments of the St2 and Y subgenome sequences, supporting the opinion of some researchers about the common origin of these subgenomes, in particular through the mechanism of recurrent hybridization (Liu et al., 2022).

Quite unexpected is the lower variability and, at the same time, relatively high species specificity among the intron sequences of the studied gene compared to the exons. This means that a detailed phylogeny of genera sharing a common and relatively ancient, yet geographically and genetically differentiated, St subgenome will still yield many surprises.

## Conclusion

Our assembled living collection of all representatives of the StY genomic group from Russia allowed us to identify, at a first approximation, the features of the phylogenetic relationships between taxa. Information on reproductive relationships, heritability of diagnostic traits, and microevolutionary relations became the basis for a step-by-step revision of the currently accepted taxonomic model of the genus Elymus (Tzvelev, Probatova, 2019). A broader understanding of species was used to construct the taxonomic model. The proposed model differs from the currently accepted one in that it takes into account the fundamental properties of taxa at all ranks: 1) the genomic constitution of species, as the basis for distinguishing and identifying independent genera; 2) the reproductive properties of hybrids, as an indicator of genetically determined interbreeding relationships; 3) phylogenetic relationships revealed using modern molecular and cytogenetic technologies. We consider the integrity of a species within the scope of a single microevolutionary complex, taking into account relationships with closely related taxa capable of exchanging genetic material with subsequent stabilization of sexual reproduction.

## Conflict of interest

The authors declare no conflict of interest.
